# Ten years of *AoB PLANTS* the open access journal for plant scientists: inception and progress since 2009

**DOI:** 10.1093/aobpla/plz025

**Published:** 2019-05-10

**Authors:** Michael B Jackson

**Affiliations:** School of Biological Sciences, Bristol Life Sciences Building, University of Bristol, Bristol, UK

**Keywords:** Annals of Botany Company, *AoB PLANTS*, botanical journals, open access publishing, Oxford University Press, publishing history

## Abstract

*AoB PLANTS* is a not-for-profit, open access, plant science journal and one of three peer-reviewed journals owned and managed by the Annals of Botany Company. This article explains events and thinking that led to the starting of *AoB PLANTS* and how the unique features of the Journal came to be formalized prior to its launch in September 2009. The article also describes how the Journal’s management developed over the first 10 years and summarizes the Journal’s achievements in a decade where open access journals have proliferated despite subscription journals continuing to dominate the publishing of peer-reviewed botanical science.

## Teaser

The peer-reviewed open access plants science journal *AoB PLANTS* is 10 years old. To mark this milestone, the founding editor of the Journal sets out the reasons *AoB PLANTS* was started and charts the highs and lows in the planning process that lead to its opening in October 2009. The Journal’s subsequent progress during a decade of unprecedented change in science publishing is described and the prospects for its future development and success assessed.

## Introduction

*AoB PLANTS* was founded by the Annals of Botany Company (Website 1), an educational charity hereafter referred to as the Company. This article reviews events leading to the launching of the Journal and the progress made since it opened in late 2009. The Company is much more long-standing. It was created in 1903 with a membership of 10 to own and manage the general plant science subscription journal *Annals of Botany* founded 16 years earlier at the personal expense of eight prominent British botanists (Jackson 2015). *Annals of Botany* remained the Company’s principal responsibility for its first 106 years. This straightforward arrangement changed with the founding of *AoB PLANTS* (Website 2). The present article evaluates the circumstances that led to this departure from past ways and relates something of the uncertainties that inevitably accompanied the decision by a small philanthropic organization to introduce a new journal into a highly commercialized international market. The account also details the initial concept and design of *AoB PLANTS* and assesses progress made over its first 10 years in the face of a stream of newer open access titles, many of which seem of questionable virtue (Butler 2013).

Despite an initial hesitancy, documented below, the experience gained from starting *AoB PLANTS* emboldened a further broadening of the Company’s activities. In 2010, it initiated the *AoB Blog*, a continuously updated online botanical digest, recently renamed *Botany One* (Website 3). By 2017, this had been expanded to include a related electronic newsletter entitled *The Week in Botany*. More recently, in 2018, a third peer-reviewed journal, *In silico Plants*, was launched (Website 4). The founding of *AoB PLANTS* therefore initiated the Company’s transition from a one-journal enterprise into a botanical publishing house owning and managing five titles. This turning point radically affected how the Company would interpret and implement its constitutional remit as it sought to serve and match, in spirit and in enterprise, the kinds of experimental plant science it most wished to support.

### Two external influences prompting the starting of *AoB PLANTS*

#### The situation at Annals of Botany

In the 10 years from 1996, manuscript submissions to *Annals of Botany* had risen more than 4-fold. This strong upward trend was accompanied by a substantial rise in the proportion of papers being turned-down. Over these years, the policy of Annals of Botany’s Chief Editor (M. B. Jackson and author of the present article) had been to increase selectivity as submissions grew. Thus, by 2006, 75 % of submissions were being rejected and, a year later, this figure had risen to almost 80 % (Jackson 2016). This not only raised scientific standards but also kept the size and cost of the Journal in line with Company-agreed limits. In one sense, the situation was pleasing since the journal was obviously doing well. However, disquiet over the policy was voiced at a meeting between the Company’s Executive Committee and Chief Editor held at Exeter University in late in 2006. The unease related to the Company’s constitutional remit to support the science of botany. With this in mind, the issue was whether *Annals of Botany* should be turning its back on so many potential authors. This concern was heightened by the Chief Editor’s opinion that many of the rejected papers contained decent science worthy of publication in principle. Similarly, since the Company operated under Charity Commission for England and Wales rules, rejecting most of the material it received fitted uncomfortably with the Commission’s ‘public benefit requirement’ viz. that the Company ‘*must benefit the public in general, or a sufficient section of the public*’ (Website 5). The 2006 Meeting concluded that the mission of the Annals of Botany Company would be better served by trying to publish more papers rather than reject ever-more papers. The choices were either to publish extra issues of *Annals of Botany* or to start another journal. The second option was decided upon and the Executive Committee (M. D. Bennett, J. S. Heslop-Harrison, R. Hunt) resolved to prepare a concept note for a new web-based, open access journal and think of a suitable title prior to putting their case to the whole Company.

#### The trend to online, open access publishing

The decision by the Company to adopt an open access model for their new journal was influenced by a growing desire more generally for scientific results to be freely available without charge to readers and for something to be done about the lucrative hold on scientific publishing by profit-hungry international companies. Many of their subscription journals appeared to give poor value for money, with the actual cost of individual journals to university and institutional libraries bearing little relation to the number of pages published or to journal Impact Factors (White and Creaser 2007). Open access journals address many of these irregularities by shifting the burden of paying from subscribers to authors in a transparent and readily accountable way, a burden that could be minimized by non-profit organizations such as the Annals of Botany Company. This approach sat well with the Company’s constitutional remit to support botanists and not shareholders. The case for free public access to taxpayer-funded research originated many years earlier in ideals incorporated into the United Nations Universal Declaration of Human Rights (1948). This was much expanded-on by several august international gatherings, most notably in 2002 and 2003 (for example, see Websites 6, 7 and 8). Although several open access journals with botanical content were already in-being by 2006 (e.g. *PLOS Biology*, *PLOS ONE*, *BMC Plant Biology*, *BMC Plant Methods*), the Executive Committee agreed to adopt the open access model for its new journal in the belief that the underlying ideals would have wide appeal but especially for younger scientists.

### Preliminary work

Six-months-on from the initial decision to start a new journal, the first concept note emerged **[see****Supporting Information—File S1****]**. This came not from the Executive Committee but from the Chief Editor of *Annals of Botany*. This was appropriate since his editorial policies were responsible for Annals of Botany’s high rejection rate. The concept note gave the new journal the interim title of *eBotany* and sought to establish aims and scope that overcame concerns about how it would distinguish itself from *Annals of Botany* and also avoid being seen simply as a cascade journal for the parent title. The concept note established the notions of a journal encompassing the breadth of botany and emphasizing excellence of execution of the science as the main selection criterion rather than considerations of broad interest, degree of originality and similar subjective issues. The note was submitted to the Executive Committee in June 2007 and to a senior journals editor at Oxford University Press (OUP) the publisher of *Annals of Botany*. OUP showed interest and this encouraged the Executive Committee to convene a special meeting at the Langham Court Hotel, London on 17 July 2007 to discuss the concept note. The outcome was cautious approval, with the Chairman and Vice-chairman of the Company retaking responsibility for the project and for putting it to the Company as a whole at its Annual General Meeting (AGM) 2 days later. Opinions at the AGM were mixed. Nevertheless, the meeting gave it guarded support and recommended further consultations with OUP by the Vice-chairman (J. S. Heslop-Harrison) followed by a planning meeting of the whole Company in November/December 2007. But doubts persisted, no detailed plans emerged from the Chairman or Vice-chairman and the intended November/December planning meeting did not take place. The hiatus may have reflected uncertainty about the wisdom of founding a new journal. Certainly, there were worries about the possible costs for the Company and whether botanical science generally was sufficiently well-funded for authors to pay to publish; the former concern being tempered by the Company’s strong financial position (Jackson 2016). For whatever reason, the project had stalled.

### Revival

The idea of starting a new journal was resurrected in April 2008, when Annals of Botany’s Chief Editor submitted a second more detailed concept note that included proposing *PLANTOME* as the title. This triggered a meeting between the Chief Editor (M. B. Jackson) and Executive Committee a month later at Cambridge. Although the name *PLANTOME* was not much liked, it was agreed that the Company’s Secretary (R. Hunt) would draft a preliminary business plan and that the Chief Editor would reopen discussions with OUP and expand the concept note for consideration by the Executive Committee on 30 June 2008. This more positive stance by the Executive Committee may have been influenced by the recent entry of the open access journal *BMC Plant Biology* into the top 20 plant science journals based on Institute for Scientific Information (ISI) Impact Factor. For whatever reason, a greater urgency now characterized proceedings. Within a month, OUP had e-mailed a market research questionnaire to ~6000 plant scientists drawn from the *Annals of Botany* user database with a participant’s prize draw worth £300 helping to achieve a 19 % return. By this time, the name *AoB PLANTS* had been agreed and it came to be understood at the Cambridge meeting that Annals of Botany’s Chief Editor would relinquish his position in the coming months to become *AoB PLANTS*’ first Chief Editor.

At the scheduled June meeting, the Executive Committee made minor adjustments to the Chief Editor’s concept note and put it to a full meeting of the Annals of Botany Company at its AGM in London on 2 July 2008. At that meeting, OUP reported that replies to the Questionnaire were broadly favourable but chose to defer a decision on publishing *AoB PLANTS* until a full analysis was complete. It was left that OUP would clarify its stance once the Questionnaire was assessed fully and that the Chief Editor would prepare a presentation for a special meeting of the full Company in October or November 2008.

By the end of September 2008, both a favourable analysis of the questionnaire by OUP and a draft business plan prepared by the Company Secretary (R. Hunt) were in the hands of the Executive Committee. The Chief Editor had also secured verbal assurances from OUP that it was indeed prepared to publish *AoB PLANTS* and that the title would remain the exclusive property of the Company. OUP had also accepted most of the technical and promotional requirements set-out by the Chief Editor and also agreed to share overt production costs such as editorial office work, website creation and maintenance, archiving and promotional work as well as any income. Actual financial splits were to be finalized later. A special meeting of the Executive Committee and the Chief Editor was then held at the Cotswold Lodge Hotel, Oxford, on 6 October 2008. Preliminary layouts for the home page, ancillary pages and scientific content were approved as were details concerning the Journal’s remit, scope and ambition. The intention to create a top-tier Advisory Board and a separate international Editorial Board was supported and OUP’s initial financial projections were well-received and incorporated into the Company’s business plan. To encourage submissions in the early years, it was resolved that author charges would be low or annulled with the Company and OUP between them standing the losses. An Extraordinary General Meeting (EGM) of the whole Company was set for 2 December 2008 when full details would be up for approval. At the time, it was intended that day-to-day editorial administration, manuscript handling etc. for the new journal would run alongside those of *Annals of Botany* at its new home at the University of Leicester, UK. But these did not materialize. Instead, an OUP Virtual Office setup was adopted. This would be run from Scotland by Dr Lulu Stader (the Editorial Assistant) who had a strong biological background. The new journal would thus be a stand-alone operation managed and operated online by its Chief Editor and Editorial Assistant backed-up by OUP.

### Company approval and a further delay

Agreeing a contract with OUP first required approval by the Company. This was expected at the December 2008 EGM. But, despite accepting the business plan and signing-off much organizational detail, there was further hand-wringing. Ever-concerned to adopt best practice and ensure value for money, the Company required the Secretary and Chief Editor to seek offers from at least one other publisher. Within 10 days, the Chief Editor met with representatives of the newly formed Wiley-Blackwell company at their Oxford Office and received a very positive response to *AoB PLANTS*. However, at a second meeting in Bristol on 12 January 2009, Wiley-Blackwell were unable to accept a low-cost article processing charge (APC) for authors; their policy being to charge full market price from the start. The impasse proved unsurmountable and, on 16 January 2009, Wiley-Blackwell backed-away. The Company Secretary and Chief Editor were unwilling to lose further momentum by turning to a third publisher since good progress was being made with OUP who, spurred-on by potential competition from Wiley-Blackwell, quickly accepting the principle of a cost-free period for authors. The Company Executive therefore decided to seek to contract with OUP. Two and a half years after the Journal was first conceived an agreement with OUP was finally signed on Thursday 17 April 2009. By then, final designs for the website, and its scientific content (see Fig. 3 for the final outcome). An Advisory Board comprising Sir Ghillean Prance (Reading), Jim Peacock (Canberra), David Beerling (Sheffield), Rens Voesenek (Utrecht) and Jerry Baskin (Kentucky) was appointed and, by October 2008, 19 Editorial Board members drawn from nine countries had also been signed-up.

### Features of *AoB PLANTS* at its inception

To encourage submissions, APCs were to be waived for the first full year of operation and raised gradually over the next 5 years to £750 ($964). To encourage speedy evaluation of submissions, editorial board members would be paid £25 ($32.3) per manuscript while referees would be offered £30 ($39) in Amazon vouchers for each paper they assessed. An initial plan to graduate referee rewards according to the speed with which their assessments were returned was abandoned. Review articles were encouraged by offering invited authors £250 ($322) for their article when published; an editor with specific responsibility for recruiting review authors being appointed. To speed-up time taken for initial rejections and lighten the load on Editorial Board members, submissions were first assessed by the Editorial Assistant and the Chief Editor against a set of published preliminary criteria of acceptability. Approved papers only would then be passed to a member of the Editorial Board to arrange peer review. These second evaluations were made against a further set of acceptance criteria that emphasized the soundness of the science. By 2016, the Journal was hoping to publish 190 papers a year with an estimated rejection rate of 55 %. Other features included double-blind refereeing, i.e. referees’ identities being concealed from authors and authors’ identities from referees. This policy was rare amongst plant science journals but offered some protection to those worried about conscious or unconscious bias linked to their identity or place of origin. Overall, the intentions were to be fast, fair, inclusive and as transparent as possible with subjective elements of decision-making being minimized. Internal communications were encouraged by the creation of a Listserv bulletin board (‘The Plants-Pub’) with Advisory Board, Editorial Board, editorial staff, Company members and relevant OUP staff as subscribers. A monthly newsletter was distributed in this way by the Chief Editor.

**Figure 1.**
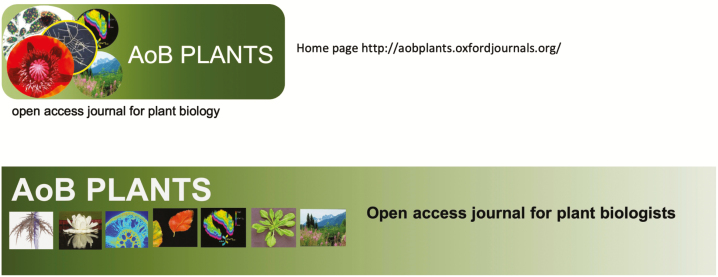
Initial designs for letterhead and webpage headings for *AoB PLANTS.* They were intended to illustrate the wide botanical coverage of the Journal and to make clear that the Journal was open access.

**Figure 2.**
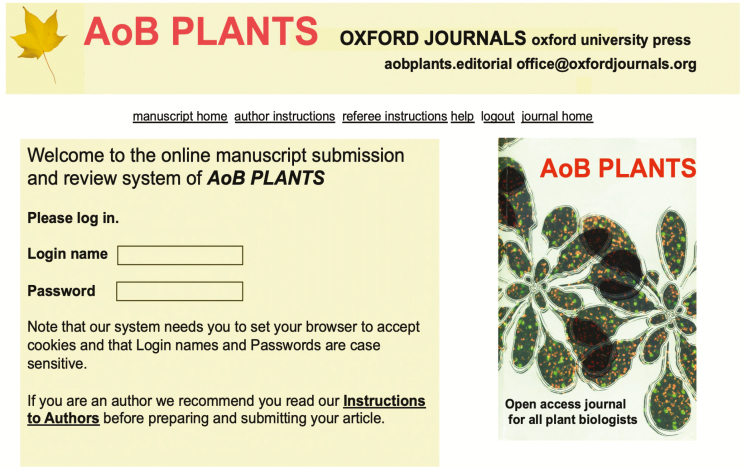
Layout for the log-in page of the *eJournal Press* manuscript submission and review website. It aimed for an uncluttered functional image in keeping with the Journal’s wish to offer a fast and efficient publishing service for authors.

**Figure 3.**
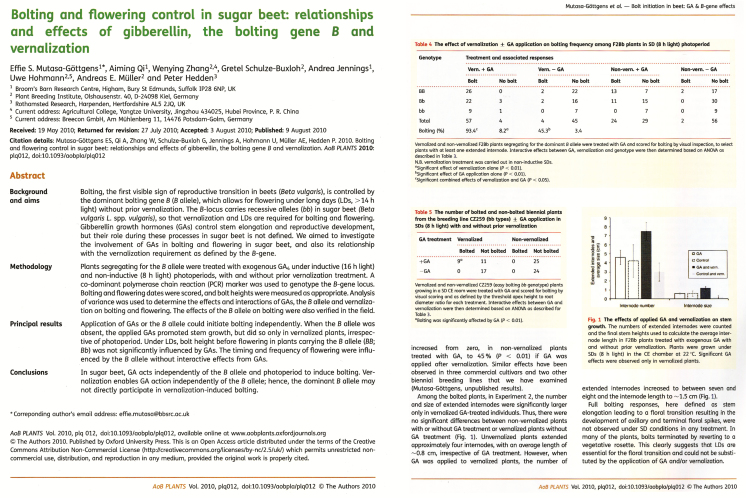
Typical layout used for papers published in *AoB PLANTS* from 2009 to 2013. To maximize clarity and visual appeal the design used colour and space in ways that would be uneconomic for a printed journal.

Earlier vivid designs for a visual identity (Fig. 1) were abandoned in favour of a subdued and less cluttered look. This included an angled sycamore leaf logo (Fig. 2, top) and an impressionistic *Arabidopsis thaliana* rosette for the faux-cover image (Fig. 2, right). The layout for fully formatted papers utilized inherent advantages of online-only publishing to create a spacious design that made restrained use of coloured text and figures, and table-blocking to enhance clarity and readability (Fig. 3). A newly released easily readable font (FontSmith ‘Me’) was adopted and, to help readers grasp the essence of each paper, abstracts were structured as mini papers up to 300 words long with section headings such as ‘Background and aims’, ‘Methodology’, ‘Principal results’ and ‘Conclusions’. Authors would also be required to end the manuscript with a brief ‘Conclusions and Forward Look’ to highlight authors’ claims of scientific worth. Plans to publish written comments on each paper by the referees and readers were not followed-through. To highlight content and facilitate access to published papers, their titles, authorship, 30-word summaries and an appropriate thumbnail image were to be displayed on the Journal’s home page. Speed of publication was prioritized by publishing authors’ typescripts within a few days of editorial acceptance; these being replaced as soon as possible by a fully formatted PDF version (Fig. 3) and an associated HTML version with numerous interactive links and downloadable figures. It was hoped that these features would not only appeal to readers and authors but also strengthen the case for the Journal’s prompt inclusion in Thomson Reuter’s Web of Science—a major entry point to the world’s scientific literature. Amongst other things, this required the Journal to have some ‘identifiably unique and distinguishing features’. Placement on the Web of Science would not only aid global access but would be needed for the eventual award of an ISI Impact Factor.

### The first 3 years (2009–12)

**Figure 4.**
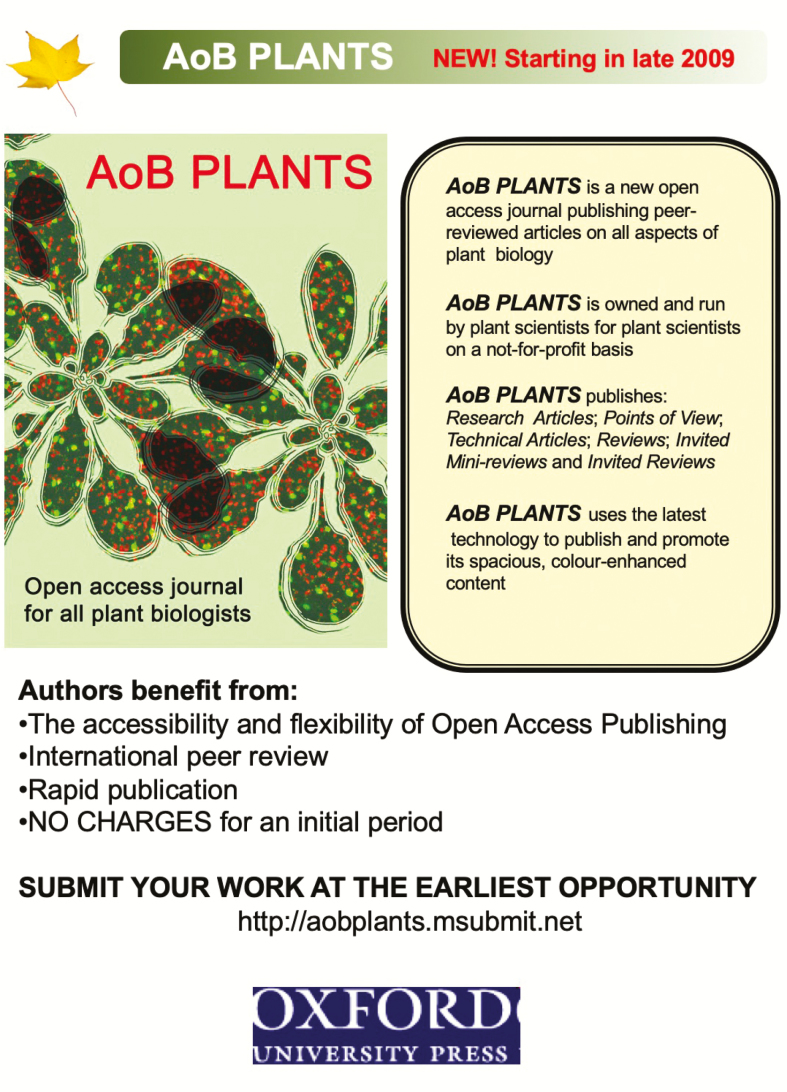
Early promotional material designed by the Journal and distributed by OUP.

**Figure 5.**
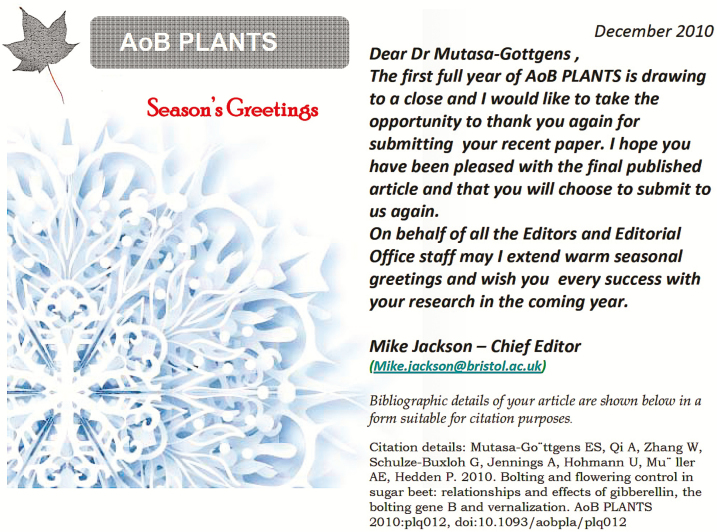
Example of e-greetings sent to authors at the end of each year. Full bibliographic details of published papers were included to help overcome any uncertainty about how articles in *AoB PLANTS* should be cited.

*AoB PLANTS* was officially launched at the Royal Botanic Gardens, Kew (UK) on 12 October 2009, the first day of a week-long conference entitled *Plant Conservation for the Next Decade.* An explanatory talk was given by the Chief Editor prior to a reception which, along with champagne and canapes, offered promotional leaflets (Fig. 4), coffee mugs, pens, Post-it Notes and memory sticks for delegates to take away. This was followed up by several weeks of intense promotional activity. Announcements were placed in newsletters of 16 plant biology societies, three mass e-mailings were made by OUP to 17 000 plant scientists worldwide and to a further 5700 recipients drawn mostly from the Annals of Botany’s database. Promotional material was distributed at two back-to-back conferences in Krakow, Poland (*Eco-physiological Aspects of Plant Responses to Stress Factors* and 4th Conference of Polish Society of Experimental Plant Biology) where student poster prizes were sponsored. Leaflets and a poster were displayed at the *Third International Symposium on Plant Growth Modeling, Simulation, Visualization and Applications* at Beijing, China. *AoB PLANTS* also sponsored a lecture generating a review article on crop trait ontology at a conference in Montpellier, France entitled *Biodiversity and Agriculture: Today’s Challenges, Tomorrow’s Research for More Sustainable Farming*. In these ways, the Journal had, by the end of 2009, comprehensively announced itself. The following year a promotional stand was arranged at the American Botanical Society’s annual meeting at Providence, Rhode Island, where an Editorial Board member (Dennis Whigham) held a ‘Meet an Editor’ session. The Journal was also promoted at the *10th Conference of the International Society of Plant Anaerobiosis*, Volterra, Italy and at the *Congress of the Federation of European Societies of Plant Physiology*, Valencia, Spain. Arrangements were made to sponsor three sessions at the 2011 *International Botanical Congress*, Melbourne, Australia and to give financial support to the 2012 *International Conference on the Plant Hormone Ethylene*, Auckland, New Zealand from which Special Issues would emerge. The botanical coverage of the Journal was extended to include phytoplankton, an editor was appointed to cover this field and promotional material was e-mailed to the phytoplankton community in collaboration with the Chief Editor of *Journal of Phytoplankton Research*, an OUP journal. In December each year, a seasonal electronic greetings card was sent to authors reminding them of the bibliographic details for their recent papers (Fig. 5). In these various ways, a pattern for promoting the Journal was established that was at the limits of what could reasonably be expected from the Journal’s small editorial team.

The first two published papers appeared towards the end of 2009. They introduced the new journal and outlined the strengths of open access publishing (Jackson 2009a, b). Six early papers in 2010 formed a Special Issue entitled *Stress and Survival in Tropical Environment* (Website 9). This was based on a session at the tropical ecology conference *Impacts of Global Change on Tropical Ecosystems* sponsored by the Journal and held in Marburg, Germany in 2009.

The success of early publicity efforts and conference promotions may be judged by comparing the Journal’s performance with expectations set out in the initial business plan. The comparison reveals a creditable rather than stellar performance. The most recent business plan anticipated four hundred submissions in the first full year (2010) with 90 actually being published. The reality was 114 manuscripts received, with 22 papers published, approximately a quarter of the number planned-for. The managerial response to this sluggish start was to postpone the introduction of author charges and to revise downwards the number of submissions anticipated. In the following year (2012) 169 submissions were received (200 were expected) but a high rejection rate meant that only 34 papers were published rather than 80 although the time taken to publish them following first submission was commendably short (15 weeks).

In mid-2011, the Chief Editor signalled his wish to stand-down the following summer. This gave the Company the opportunity to recruit internationally for a replacement and, by June 2012, Professor J. Hall Cushman a plant ecologist from Sonoma State University, California, USA had been appointed. In the months leading-up to the change in editorship, promotional activities continued undiminished. Leaflets and pens etc. were distributed at the *2011 Wetland Biogeochemistry Symposium*, Prague, Czech Republic where financial support was given for a session on *Phragmites* ecology. Promotional material was sent to the *Fourth International Orchid Conservation Congress*, Hluboka, Czech Republic and a stand was taken with OUP at the *2011 Botanical Congress*, Melbourne, Australia where three sessions sponsored by the Journal generated papers on ‘*Trace Gas Analysis*’, ‘*Pollen-pistil Interactions*’ and ‘*Flooding Stress*’. *AoB PLANTS* also sponsored the opening plenary lecture on computational modelling of development at the *2011 Conference of the Polish Society of Experimental Plant Biology*, University of Wrocław. Financial support was given to the *2011 International Symposium on Plant Biotechnology*, Birla Institute of Technology, India, the 2011 conference *Molecular basis of plant stress*, University of Plovdiv, Bulgaria 21–23, the *2012 Latin American Congress of Algal Biotechnology*, Universidad de Concepción, Chile, and the *2012 International Conference on Polyploidy, Hybridization and Biodiversity*, Zámek, Czech Republic where four student poster prizes were awarded. Finance was also given to the *IX International Conference on the Plant Hormone Ethylene* in New Zealand (2012) and to a session at the *2012 Society for Experimental Biology Meeting* at Salzburg, Austria on ‘*Chloroplast biogenesis*’. Plans were also laid to support the *2013 Conference of the International Society for Plant Anaerobiosis* at the International Rice Research Institute, Philippines from which a sizeable special issue was anticipated. E-mail publicity by OUP and the Editorial Office continued, targeting China in particular. These measures introduced the Journal to numerous countries with sizeable communities of plant biologists thereby enhancing prospects for high-quality papers in the years ahead.

### The 6 years to 2018

**Figure 6.**
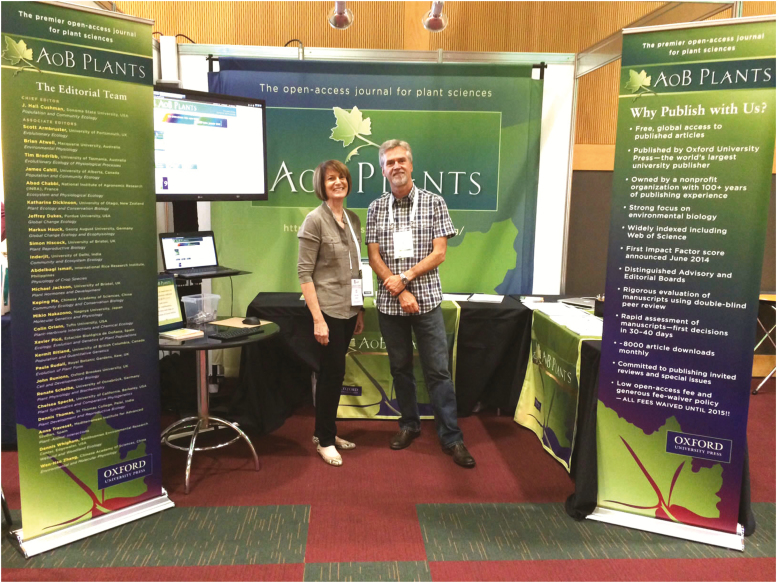
The Journal’s stand at the 2014 FESPB/EPSO Plant Biology Europe meeting at Dublin, Ireland. The Chief Editor (Hall Cushman) and the Managing Editor (Gail Rice) are shown.

Hall Cushman commenced his work as Chief Editor in July 2012 and, within a short time, overhauled most aspects of the Journal. A new image was commissioned from a Californian graphic design firm (Fig. 6) and more assertive tag lines such as ‘Next-Generation Publishing in the Plant Sciences’ and ‘The Premier Open Access Journal for Plant Sciences’ were adopted. There was also a re-focussing of subject coverage in favour of environmental and evolutionary plant biology. Procedural alterations included abandoning the overt two stage evaluation process and making a much-needed revision to the issue/volume numbering system. Scientific rigour as the main criterion of acceptability was retained. The existing Advisory Board was retired with the five replacements being environmental scientists having considerable editorial experience with other peer-reviewed journals (D. Ackerly, University of California, USA; C. Korner, University of Basel, Switzerland; D. Richardson, University of Stellenbosch, South Africa; S. Silver, Ecological Society of America; D. Simberloff, University of Tennessee, USA). The Editorial Board was also strengthened and diversified with over 30 replacement Associate Editors from eight new countries. The proportion of women editors was increased considerably. Previous arrangements to facilitate the evaluation of selected manuscripts that *Annals of Botany* previously declined on subjective grounds were improved and this formed the basis of a productive collaboration between the two journals. *AoB PLANTS*’ overall capability was increased by appointing a Managing Editor (Gail Rice) and a replacement Editorial Assistant (later upgraded to Deputy Managing Editor) to operate the Virtual Editorial Office (Dr Joanne Ferrier). Other changes included an overhaul of the Journal’s website with recent papers and their thumbnail images no longer fronting the landing page. An ‘Editor’s Choice’ feature was introduced to highlight the most promising papers and a simplified, compact PDF format for published papers was adopted. Requirements for a structured abstract and author’s ‘Conclusions and Forward Look’ were also relaxed. The existing Creative Commons Attribution Licence was liberalized to allow unrestricted use, distribution and reproduction of articles by anyone as soon as accepted, provided that authors, citation details and publisher are identified. To encourage invited review articles, payment was raised to £396 ($500) and later to £774 ($1000). These comprehensive changes were partnered with a marketing surge, spearheaded by increased conference attendances, OUP mailshots and by opening a Facebook page, a Twitter account and creating a page on Wikipedia. In addition, numerous promotional messages were sent to various society Listserv bulletin boards. Posting *AoB PLANTS* content on the *AoB Blog* (now *Botany One*) was streamlined while the Journal’s own Listserv bulletin board was replaced by an e-mailed newsletter.

In addition to the six special issues arranged up to and including 2012, a further seven with ecological themes were published in the years to mid-2018. Review articles continued to be invited as a means of attracting leading authors to the Journal and encouraging citations (20 invited reviews were published from 2013 to mid-2018 incl.). Promotional work also continued much as before with editorially staffed stands at conferences such as FESPB/EPSO Congresses and annual meetings of the British Ecological Society, Botanical Society of America and the Ecological Society of America. Flash drives with links to notable papers; pens, bookmarks and water bottles imprinted with the new *AoB PLANTS* logo were commissioned for use at these conferences along with a range of display material (Fig. 6). Article processing charges for authors commenced in January 2013 at £781 a paper ($1000) but the decision was reversed 2 months later in the face of a sharp loss of submissions. With hindsight, it was probably unwise to have started charging before the Journal was given an Impact Factor. Fall-out from this included a one-off management meeting held in March 2014 at a Boston (USA) hotel where the Company’s Chairman (H. Dickinson) and Secretary (R. Hunt) and the Chief Editor and Managing Editor of *AoB PLANTS* met to discuss how to reverse the 2013 setback and secure 170+ published articles per annum by 2017. A new publicity surge was agreed as was the appointment of a Social Media Editor (Lara Reichmann) with the remit to maintain and develop content for Facebook and Twitter, the *AoB Blog* and OUP sites whilst also developing new features for the *AoB PLANTS* website.

### Outcomes

The extensive changes and publicity surge brought-in by Cushman with support from the Company Executive came at a cost. Annual expenditure in 2013, 2014 and 2015 was three times that for the first full year of operations (2010). However, the expense paid-off with submissions rising 300 % during 2014 and 2015. In later years, inevitable cost-cutting brought expenses down a little although they still exceeded those of 2010 almost two and a half times. Despite strong arguments against reintroducing author charges voiced by the Chief Editor, the Company Executive and OUP decided to recommence APCs in January 2016. The starting fee was £781 ($1000) and this was expected to grow annually to £1523 ($1950) by January 2020. These fees were below those charged by most other open access plant science journals but slightly above that charged by *PLOS One*, a general science journal publishing numerous plant biology papers. Full or partial fee waivers would be given to authors from developing countries, and for selected special issues and invited reviews. To help keep fees low, some economies were necessary. These included abandoning the recently created position of Social Media Editor, stopping incentive payments to associate editors and no longer rewarding referees with Amazon gift vouchers. Bringing-in author charges reduced submissions by about a third over the next 2 years; the effect being much smaller than that experienced 3 years before. Prior to the advent of APCs in 2016, the number of papers published annually had risen almost 7-fold compared with the Journal’s first full year (2010) but, following the introduction of APCs submissions halved. Making *AoB PLANTS* both grow in volume and pay its way was clearly going to be a challenge but, for the foreseeable future, the Company was prepared to fund the deficit as part of its constitutional remit to support botanical science.

After some initial reluctance, support from OUP to publicize *AoB PLANTS* was expanded considerably from 2015, especially electronic publicity for China. This resulted in an increasing proportion of published papers from that country (11 % in 2016 and 14 % in 2017), exceeded only by the USA (18 % in 2016, 34 % in 2017). OUP’s promotional efforts also included numerous e-mail shots such as an ‘Author and Reviewer Thank you’ campaign, a ‘Ten Reasons to Publish in *AoB PLANTS*’ campaign and a ‘Highly Cited’ promotion. The Journal was thus enjoying unprecedented support from OUP’s publicity team, an outcome of much lobbying by the Company Chairman and Secretary and the Chief Editor. This heightened support may also have reflected a recognition by OUP that bringing-in author charges required them to initiate compensating support measures.

In 2014, recognition of the Journal’s academic standing arrived in the form of its first Impact Factor from Thomson-Reuters (formerly Institute of Scientific Information and now Clarivate Analytics). The initial rating, covering citations to papers published in 2011 and 2012, was 1.743. This placed *AoB PLANTS* 72nd out of 196 plant science journals. In the following year, the Impact Factor rose to 2.73 and, by 2017, it had reached 2.821 placing *AoB PLANTS* amongst the top 25 % of plant science journals and above traditional competitors such as *American Journal of Botany* and *International Journal of Plant Science*. A listing was also secured in the Environment/Ecology category with *AoB PLANTS* positioned 51st amongst 158 journals. Thus, in its first 10 years and on the basis of Impact Factor, the Journal had overtaken numerous long-established plant science titles and outperformed most of the more recent open access upstarts. The trend in online journal usage, measured in terms of full text downloads, was also healthy. Based on 2010, the first full year of the journal’s operations, downloads had grown 4.5-fold by 2017, while interest in the Journal in terms of requests for e-mailed tables of content rose more than 6-fold. This buoyant picture was complimented by some exemplary service to authors in terms of the time taken to process submissions. Time to provisional acceptance of submitted papers in the first full year of operations (2010) was 52 days but, by 2017, had been shortened to 46 days. By 2018, the average time taken to decide the fate of all submissions was steadily decreased to about 30 days. The time for accepted papers to appear online was maintained throughout at 7–11 days while the time need to convert these to fully formatted HTML and PDF versions was consistent over the years at 5–7 weeks with some recent papers taking only 2–3 weeks. Overall, authors had seen the time taken to publish their papers from first submission slow slightly from just under 4 months in 2010 to 4.5 months in 2017 as a result of authors’ revised papers being scrutinized with increased rigour. A strength of the Journal throughout has been its broad geographical coverage. In 2010, first-author countries of origin numbered 13 but this had grown to 38 by 2015 with submissions that year being drawn from 52 countries. Currently, papers from the USA (24 %), Germany (14 %) and China (10 %) lead the way.

In October 2017, the Chief Editor announced his wish to step down. Hall Cushman’s legacy would be a smooth-running journal with fair and transparent decision-making procedures for authors, a large and highly international editorial board, a strong and rising Impact Factor and, since 2016, one now generating income from author charges. The challenge for the future would be to build on the reputation for quality science and excellent service to authors and readers while scaling-up the operation sufficiently to recompense the Company and OUP for their original and ongoing investments. By the end of 2017, the total costs incurred had exceeded £600 000.

### 2018 and beyond

**Figure 7.**
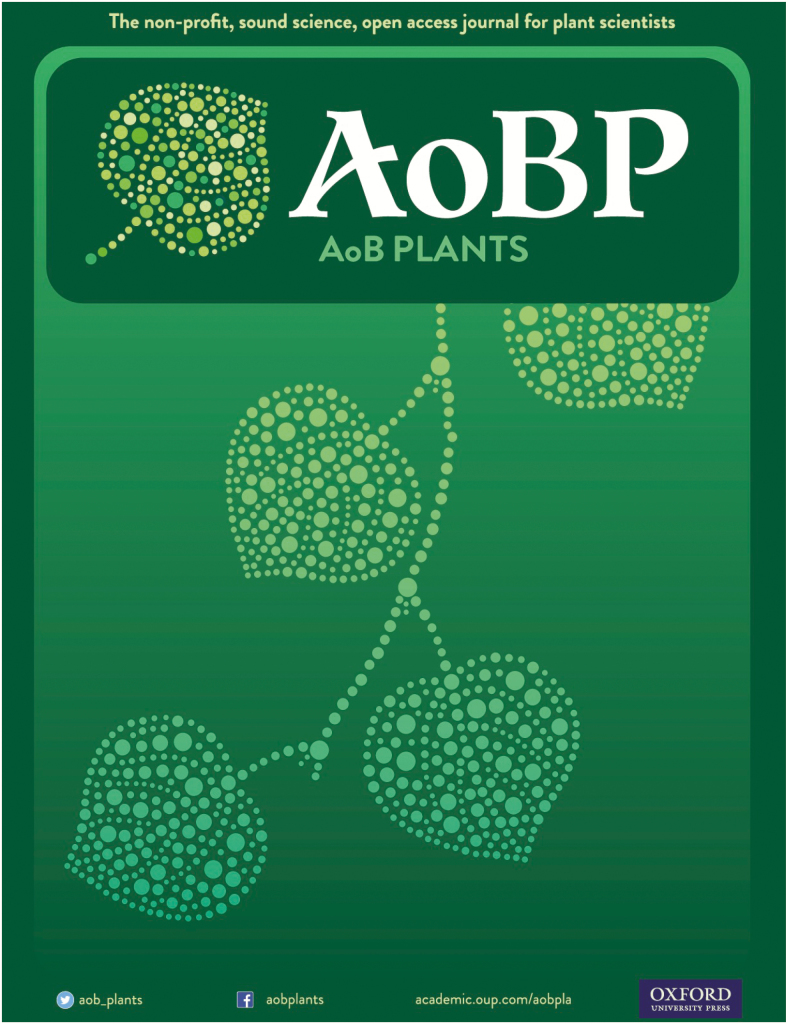
The 2018 new look for AoB PLANTS. This image change sought to foster ‘AoBP’ rather than ‘AoB PLANTS’ as the Journal’s everyday name.

Hall Cushman’s resignation led to the appointment, in January 2018, of Dr Thomas N. Buckley, from University of California, Davis, USA as the Journal’s third Chief Editor. Buckley was sympathetic to the original ideals of the Journal, especially its broad botanical coverage and the avoidance of subjective opinions as principal criteria of acceptability. By June 2018, Buckley’s plans for the journal’s look and ways of working were already being implemented in co-operation with the Company’s Executive Committee and OUP. The initial goal was to increase submissions. To this end, a publicity drive was organized and conference attendances arranged. In addition, a top tier of five decision editors would be incentivized by linking their honorarium payments to submission rates. This would necessitate reducing the Chief Editor’s own honorarium and eliminating the expense of a managing editor once an initial 12/18 months reorganization period was over; the Chief Editor taking-on this role thereafter. The five decision editors were to be ‘Section Chief Editors’ separately responsible for attracting papers, allocating associate editors and making final decisions in the areas of *Agroecology & Environment*; *Evolution & Diversity*; *Plants, Ecosystems & Climate, Form and Function*; *Populations & Communities* using acceptance criteria clearly set-out on the Journal’s website. Additional areas would be added later. An overarching consideration was that ‘*all good data and sound arguments deserve a voice*’. To encourage acceptances and journal loyalty, Buckley also reinstated financial rewards to Associate Editors and increased payments to £40 ($52) per manuscript. The post of Social Media Editor was also restored and, to signal regime change and the accompanying organizational and attitudinal shifts, the Journal’s image was remodelled once more (Fig. 7). In a notable departure from past practice, post-publication reviews of papers would be encouraged. In this way, the Journal’s content would be improved after publication by allowing readers to post comments and giving the opportunity for authors to update their research. These changes amounted to much more than simple rebranding which, by itself, is often poorly rewarded (Clark 2018).

## Conclusions and Forward Look

*AoB PLANTS* was born out of burgeoning submissions to its sister journal *Annals of Botany*. It was also founded at a time when open access publishing of scientific research was being seen a promising a way out of mounting difficulties with the prevalent subscription-based model. These difficulties hinged on unprincipled access barriers to scientific data, unhelpful copyright restrictions and perceived excessive profiteering by some dominant international publishers. Ten years later however, the subscription model remains dominant and open access publishing continues to spark controversy (Monbiot 2018) whilst also spawning a host of new open access journals. Against this challenging background *AoB PLANTS* has successfully established itself as a journal maintaining high standards of scientific integrity, transparency and fairness and one well-positioned to benefit from the decision by several large European science-funding organizations to mandate open access publishing of the science they pay for (their ‘Plan S’—Website 10). The Journal has recently revamped its operations under the guidance of a new Chief Editor who believes strongly in the ideals of open access publishing. It is too early to assess the full impact of these recent developments on the performance of *AoB PLANTS* but at the time of writing, the latest changes had started to prove their worth. After reaching a low point in June 2018, submissions rose steadily and, by January 2019, had grown by 75 % compared to the previous year’s nadir.

Future success for *AoB PLANTS* will rest on the inherent appeal of a well-run, not-for-profit journal that is owned and managed by plant scientists for plant scientists and has no fiscal demands from shareholders or investors to add to authors’ costs. These features and its timely and transparent evaluation procedures are backed-up by OUP, the world’s largest and oldest university publishing house that also espouses a not-for-profit ethos. This raft of attributes makes *AoB PLANTS* an increasingly persuasive and appealing prospect for authors and readers alike.

## Supporting Information

The following additional information is available in the online version of this article—

File S1. Initial concept note for the new on-line open access Journal.

plz025_suppl_Supporting_Information
