# Supplementary material for: Ten years of AoB PLANTS the open access journal for plant scientists: inception and progress since 2009
Source: AoB Plants. 2019 May 10;11(3):plz025. doi: 10.1093/aobpla/plz025 (PMC6524486; doi:10.1093/aobpla/plz025)
Supplement: plz025_suppl_Supporting_Information [file plz025_suppl_supporting_information.pdf]

Supporting Information

Initial concept note for the new on-line open access Journal (July 2007)

Submitted by the Chief Editor *of Annals of Botany* (M.B Jackson) to the Executive Committee of the Annals of Botany Company on 16 July 2007 at a meeting held at the Langham Court Hotel, London.

Some possible titles:

Botany One; Botany Focus; Botany Direct; Botany First; eBotany ; eBotanyOne; Botany Journal; Botany Online; Botany Open; Plants; Plant Progress; Plant BioScience

Suggested banners and slogans:

eBotany – turning results into progress

eBotany – putting good scientific practice first

Journal Description

eBotany is an online, internationally peer-reviewed plant science journal. The Journal is entirely open access, levies no charges to its authors (initially) or to readers and publishes full-length research articles, reviews, short communications and comment papers on all aspects of plant biology. ***eBotany*** is produced by the Annals of Botany Company in conjunction with Oxford University Press. Both are not-for-profit organisations that seek to promote information flow across the plant science community.

eBotany will be mounted on the OUP/High Wire Press system and on PubMed Central. The journal is fully searchable, linked electronically to the wider scientific literature via CrossRef, Medline and the ISI Web of Science and will be covered by a wide range of abstracting services. Its unique contribution to plant science publishing **is a concentration on the excellence of the work itself** rather than on considerations of generality of interest, novelty, compliance with current opinion and similar subjective issues. The readership will decide on the usefulness of papers published in ***eBotany*** and the well-known system of e-letters will be made available for on-line comment on the papers published. These comments will remain attached in perpetuity to the article they relate to. The main criteria for acceptance are that the work is well-executed, clearly written and draws conclusions that are broadly compatible with the results presented while recognising that authors are fully responsible for their own views. This simplification allows for faster and less contentious refereeing that puts good scientific practice first. The high standards of formatting, illustrative material and graphical information to be found *in Annals of Botany* will be features ***of eBotany.***

Submission and Peer Review

Manuscripts are submitted using a dedicated on-line submission system. Submitted papers are reviewed by referees that are selected by members of an international Editorial Board. Editors and referees will be aware of the unique criteria for acceptability set by ***eBotany.*** They will concentrate on the standard of the science, the reasonableness of the conclusions in the light of the evidence presented, and the extent to which there is satisfactory integration with preceding studies. While authors can still expect to revise their original submissions prior to any final acceptance, the overall approach is designed to identify well-crafted science, promote its fast publication *and give instant access* on line once papers are accepted (i.e., prior to final copy editing and formatting).

Open Access

All accepted articles are made freely available online immediately upon publication, as part of a long-term archive, without subscription barriers to access. We have chosen to implement the Creative Commons Attribution-Non Commercial License for the articles we publish. This means that users of articles are entitled to use, reproduce, disseminate, or

display these articles provided that the original authorship and ***eBotany*** bibliographic information is fully attributed. Authors are entitled to deposit a post-print of their accepted manuscript and/or the finally published version of the article into an institutional or centrally organized subject repository, immediately upon publication. This is provided that they include a link to the published version of the article on the journal's website, and that the journal is attributed as the original place of publication, with correct citations given. Copyright is held by the corresponding author or his employer.
